# Supplementary figures and images for: Lgr5+ ductal cells of von Ebner’s glands: Candidate stem cells for turnover of posterior tongue taste buds
Source: PLoS One. 2026 Jan 23;21(1):e0340679. doi: 10.1371/journal.pone.0340679 (PMC12829810; doi:10.1371/journal.pone.0340679)

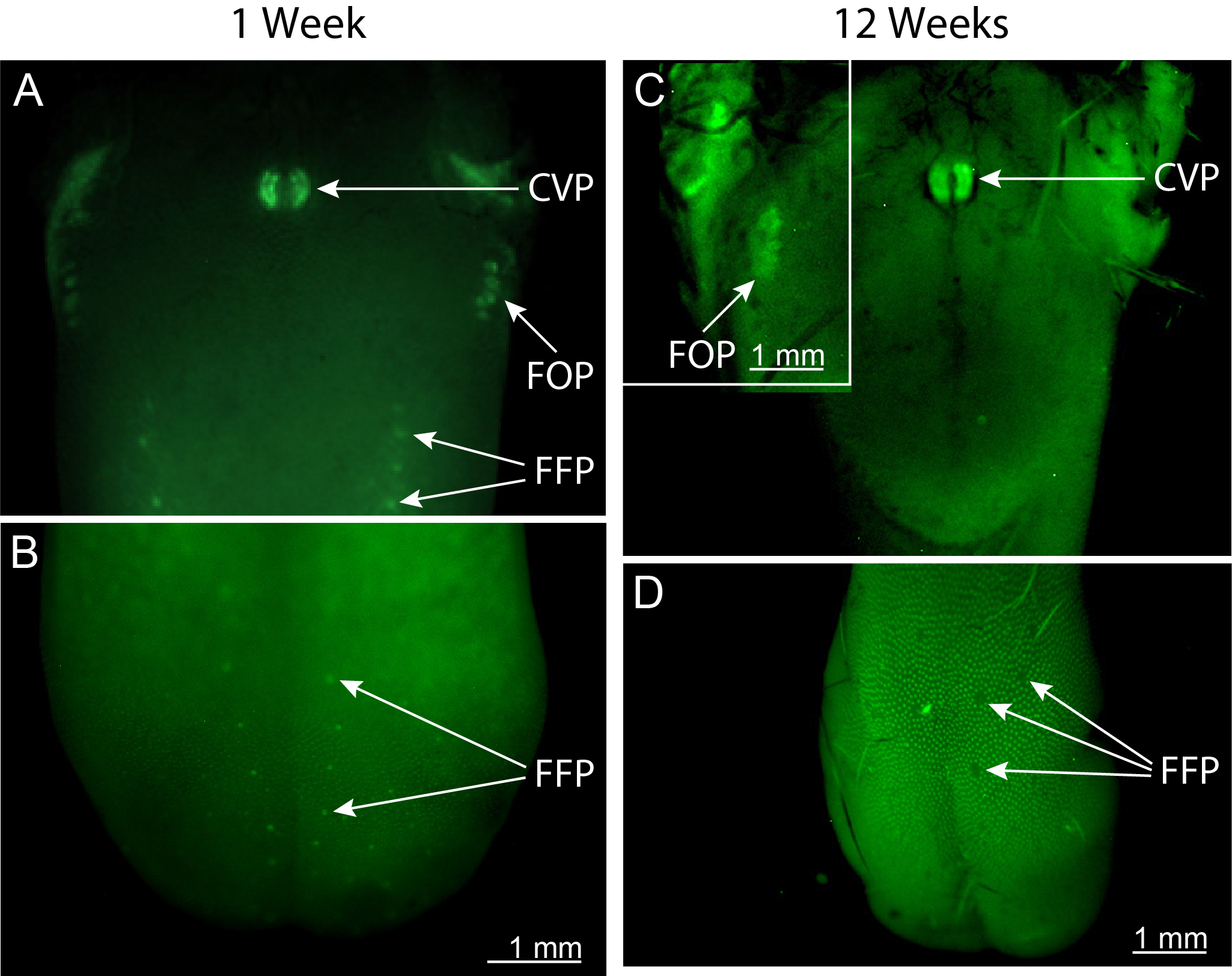

Supplement: S1 Fig — (A) and (B) 1 week. In the posterior tongue (A), GFP is localized centrally at the site of the circumvallate papilla (CVP) and laterally in the foliate papillae (FOP). Arrays of punctate label are also seen on both the posterior (A) and anterior (B) tongue at sites where single fungiform papillae (FFP) are located. (C) and (D) 12 weeks. Note that, at this time, Lgr5 labeling is maintained in circumvallate (C) and foliate (C, inset) papillae, but is no longer evident in the fungiform papillae (D). (A) and (B) are taken from a single tongue. The main images in (C) and (D) are of the same tongue, whereas the inset image pictured in (C) is from a different mouse. The scale bar in (B) applies to (A); scale bar in (D) to (C). Tongues from 8 (1 week) and 5 (12 weeks) mice were examined in these experiments. (TIF) [file pone.0340679.s001.tif]

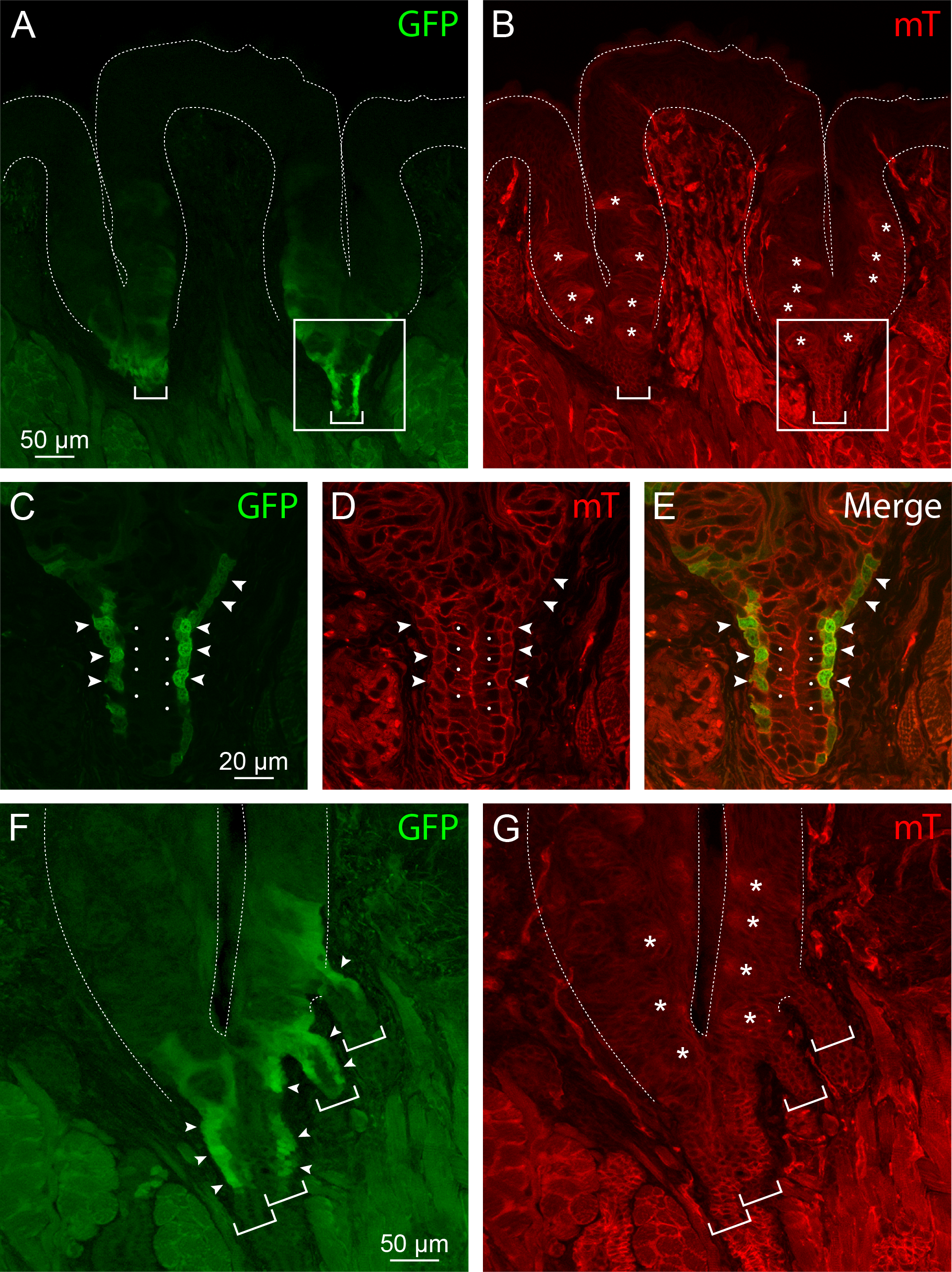

Supplement: S2 Fig — (A) and (B) Low power images of a single papilla. In (A), GFP fluorescence is concentrated at the base of the papilla on both sides. Cell outlines revealed by expression of membrane Tomato (mT), seen in the corresponding image (B), show that Lgr5+ cells in both regions are associated with excretory ducts (brackets). The plane of section passes through the center of the duct on the right and tangentially through the epithelial wall of the duct on the left. The boxed areas in (A) and (B) are shown at 2X magnification in (C) and (D), respectively. Highly fluorescent GFP+ cuboidal cells (arrowheads) occupy the outer/basal epithelial layer of von Ebner’s gland excretory ducts, while columnar cells in the inner/luminal layer of the ducts (indicated by dots) are unlabeled. (F) and (G) Intersection of excretory ducts with the circumvallate papilla. Intense GFP labeling is observed in the walls of four ducts (indicated by brackets) where they merge with the papilla. While two ducts merge at the base, the others join the papilla at more superficial levels laterally. Dashed lines indicate papilla boundaries. Taste buds are indicated by asterisks. (A), (B), (F) and (G): Maximum projection of 3 optical sections. (C-E): Single optical sections. Sections of circumvallate papillae were obtained from 6 non-tamoxifen treated mice. (TIF) [file pone.0340679.s002.tif]

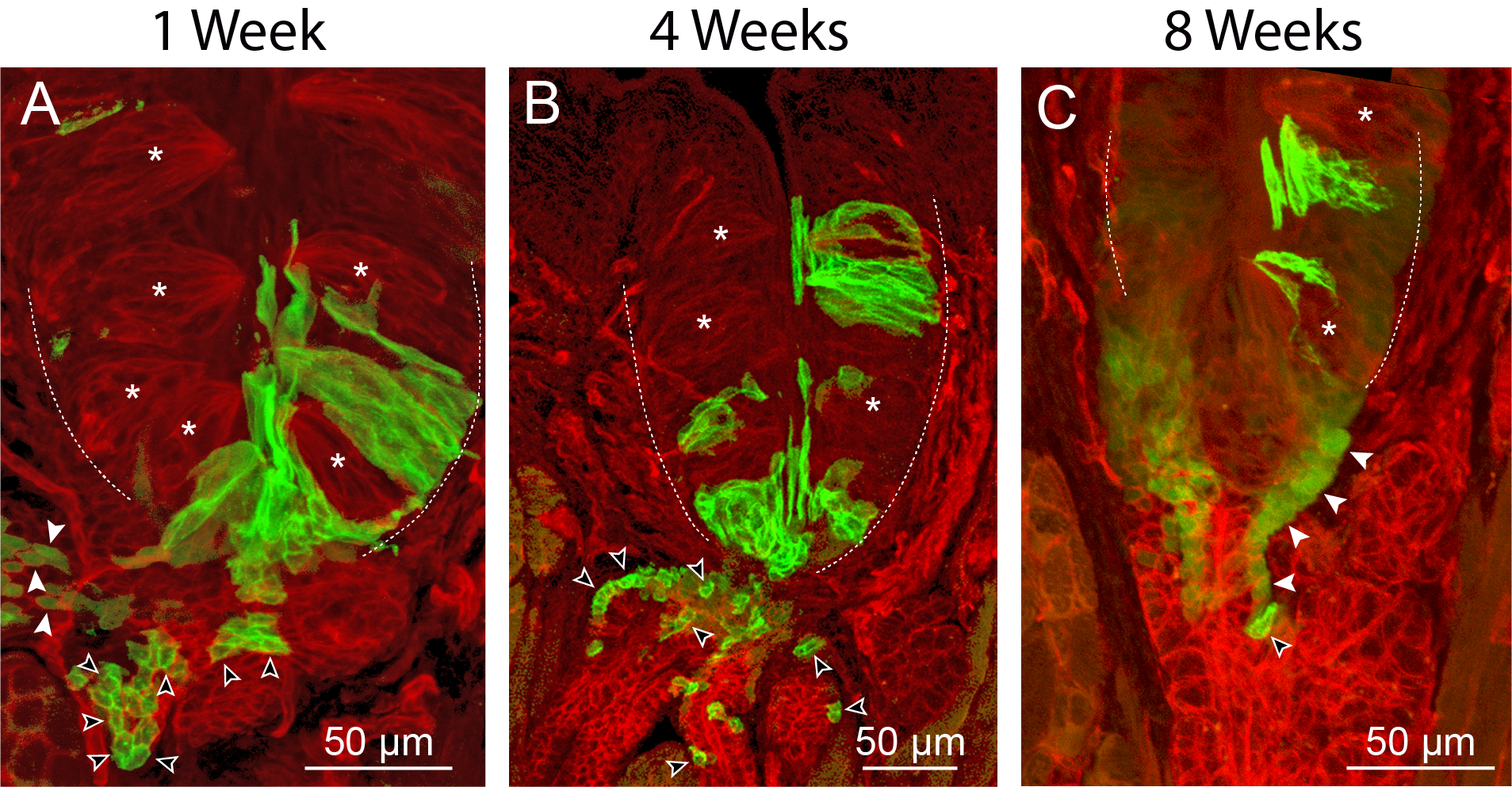

Supplement: S3 Fig — Unlike all other reported experiments, in this case Lgr5-GFP-IRES-CreERT2;R26-mTmG mice were injected with two consecutive doses of tamoxifen (days 0 and 1), and then examined at the indicated times for induced membrane GFP expression. At 1 week (A), 4 weeks (B) and 8 weeks (C), numerous membrane-labeled cells are present in and around taste buds, including in the superficial epithelial layers lining the trench. Duct-associated Lgr5+ cells, both with and without tamoxifen-induced labeling (open and filled arrowheads, respectively), are also present throughout the 8-week period, the longest time examined. Asterisks indicate individual taste buds. Dashed lines demarcate the borders of the papilla. Tissues were examined from 4 (1 week) and 3 (4 and 8 weeks) tamoxifen treated mice. (TIF) [file pone.0340679.s003.tif]

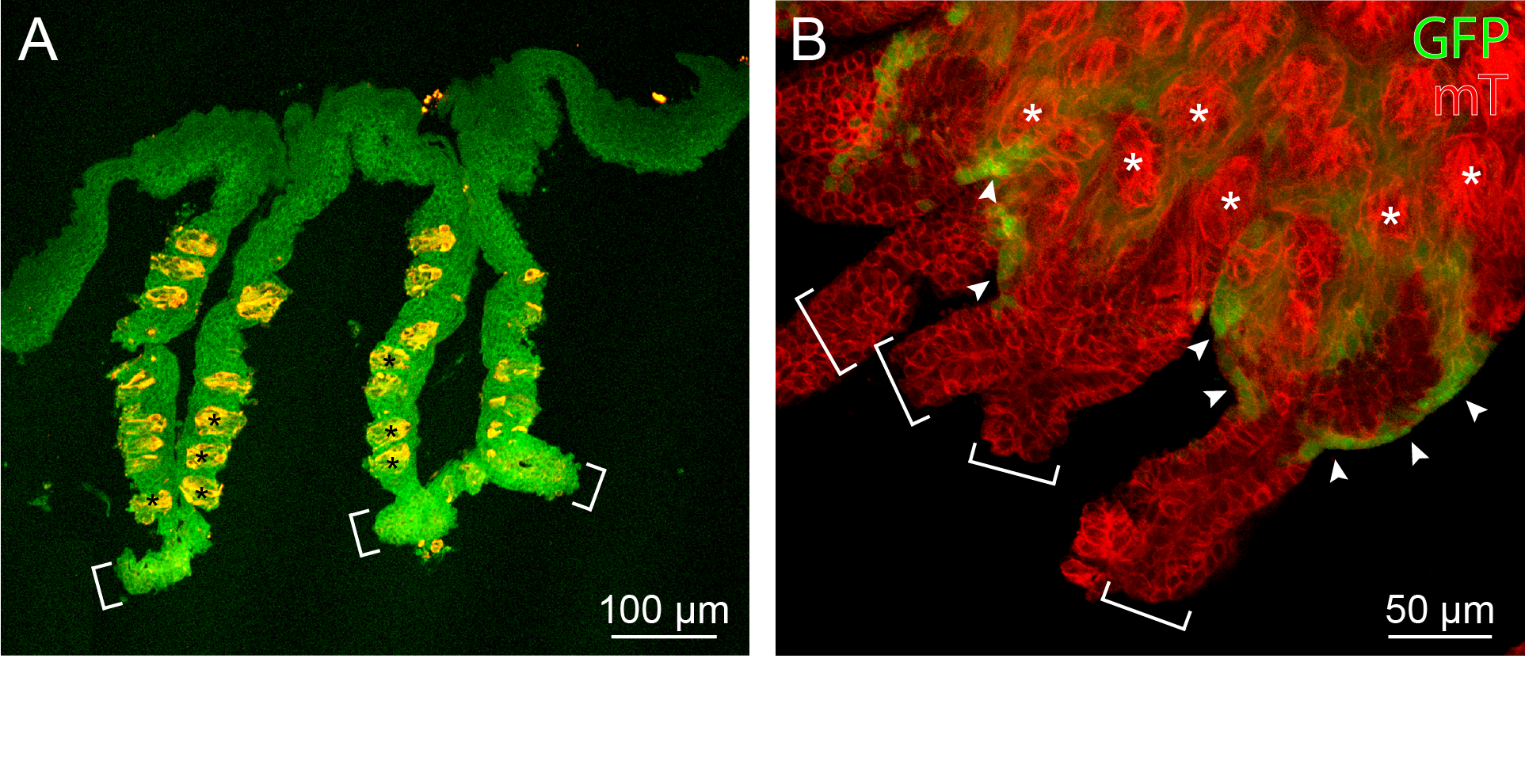

Supplement: S4 Fig — (A) Frozen tissue section from a non-transgenic animal labeled with anti-Krt8. Background tissue fluorescence (green) overlaid with Krt8 immunoreactivity (red) produces yellow-appearing taste buds (asterisks). Associated with the papilla are proximal segments of salivary ducts (brackets), which remain attached after the isolation procedure. (B) Papilla epithelium whole mount from an adult Lgr5-GFP;R26-mTmG mouse; all cells express membrane tomato (mT, red). Lgr5+ cells with cytoplasmic GFP (green) are present in the proximal excretory segments (arrowheads) of duct fragments (brackets) that partition with the excised tissue. Tissues from 3 mice were used in each experiment. (TIF) [file pone.0340679.s004.tif]
